# Intervertebral disk regeneration in a rat model by allopurinol-loaded chitosan/alginate hydrogel

**DOI:** 10.17305/bb.2022.8550

**Published:** 2023-08-01

**Authors:** Linhui Ye, Zengxin Gao, Saeed Rohani

**Affiliations:** 1 Department of Orthopedics, Nanjing Lishui People’s Hospital, Zhongda Hospital, Lishui Branch, Southeast University, Nanjing, China; 2 Department of Orthopedic Surgery, Zhongda Hospital, School of Medicine, Southeast University, Nanjing, China; 3 Department of Tissue Engineering, Tehran University of Medical Sciences, Tehran, Iran

**Keywords:** Allopurinol, intervertebral disk regeneration, composite hydrogel

## Abstract

Intervertebral disk degeneration remains one of the most challenging health problems. In the current study, allopurinol was loaded into the chitosan nanoparticles and then incorporated into chitosan/alginate hydrogels and then further studied for its disk regeneration potential in a rat model. In vitro studies were performed to characterize the hydrogel system, including scanning electron microscopy, cell viability assay, cytoprotection assay, cell migration assay, swelling assay, and drug release assay. In vivo study was performed in a rat model of the intervertebral disk injury. Animal studies showed that allopurinol-loaded hydrogels had significantly higher disk regeneration potential compared with other experimental groups. The gene expression studies showed that the animals treated with allopurinol-loaded hydrogel had significantly higher tissue expression levels of type I and type II collagen genes than other groups. Furthermore, the tissue expression levels of nuclear factor κB (NF-κB) and glutathione peroxidase (GPx) genes were significantly lower in this group. The relative expression levels of type I collagen, type II collagen, NF-κB, and GPx genes in the allopurinol-loaded hydrogel group were 2.77 ± 0.2%, 2.86 ± 0.25%, 0.58 ± 0.03%, and 0.45 ± 0.02%, respectively. We showed for the first time that allopurinol-loaded hydrogel promoted intervertebral disk repair, which could be due to its potential to modulate oxidative stress, reduce inflammation, and improve matrix synthesis.

## Introduction

The lower back pain significantly reduces the patient’s life quality and is considered to be one of the main causes of disability worldwide. Although various factors may be involved, it seems that intervertebral disk injury and its degeneration are strongly associated with the disease [1]. Current treatment modalities focus on alleviating the disease symptoms or slowing down the degeneration process. Despite being beneficial in short term, these treatments fail to restore the disk’s structure. Recently, intervertebral disk tissue engineering has shed a new light on impenetrable intervertebral disks [2, 3]. This technology tries to build tissue-engineered intervertebral disks in vitro by combinations of cells, biomaterials, and signaling cues. In this endeavor, scaffold fabrication methods play a crucial role by building biomimetic scaffolds for biological activities of the cell [4]. Indeed, the replacement of damaged nucleus pulposus by injectable hydrogel systems is gaining momentum in the intervertebral disk regeneration strategies. These scaffolds are highly similar to the nucleus pulposus extracellular matrix (ECM) and can be engineered to exhibit its similar biological features as well [5, 6]. In this context, material selection has a profound effect on the healing function of the developed hydrogel. Cells–material interactions may affect their signaling systems and ultimately result in different therapeutic outcomes.

Among various scaffolding biomaterials, hydrogels produced from chitosan and alginate have shown a promising therapeutic activity in various disease models [7]. Due to their opposite charge, these two polymers form a stable hydrogel in a process known as polyelectrolyte complexation. Furthermore, hydrogels produced from these two polymers have a high water content, an excellent biocompatibility, tailorable mechanical properties, and drug delivery potential [8]. However, the complex pathophysiology of intervertebral disk degeneration requires a versatile approach for slowing down the tissue degeneration and promoting the regeneration process [9]. Recently, the incorporation of drug delivery systems in the matrix of tissue-engineered constructs has been proposed to improve their bioactivity. In this framework, antioxidant reagents have been widely explored to modulate oxidative stress at the tissue injury site [10].

Allopurinol is an antioxidative drug that is primarily used to decrease uric acid levels. In addition, its anti-oxidative potential has been shown in previous studies [11]. In this regard, Durán Fernández-Feijóo et al. showed that allopurinol’s administration reduced oxidative stress and mitigated tissue injury in a rat model of hypoxic-ischemic encephalopathy [12]. Varrica et al. loaded allopurinol into lipid nanoparticles and used them to treat skin wounds. They showed that allopurinol-loaded nanocarriers were not toxic against HaCaT cells and significantly promoted wound closure compared with the control animals [13]. In addition, Gois et al. [14] showed that allopurinol prevented rhabdomyolysis-associated acute kidney injury through the modulation of oxidative stress. This drug can be incorporated into the matrix of chitosan/alginate hydrogel by blending with the polymeric solution. However, this method of drug loading may lead to a burst drug release and reduced bioavailability [15]. Conversely, composite hydrogel systems produced from drug-loaded nanoparticles and a hydrogel matrix provide a better control over drug release. In this regard, chitosan nanoparticles (CHNPs) have gained significant attention in pharmacology and tissue engineering applications [16]. These drug carriers are biocompatible, biodegradable and have a high encapsulation efficacy [17].

Based on these principles, the authors dispersed allopurinol-incorporated CHNPs (ALCHNPs) into the matrix of chitosan/alginate hydrogel to treat a rat model of intervertebral disk injury. This is the first study investigating the healing function of allopurinol-incorporated hydrogels in the intervertebral disk injury.

## Materials and methods

### Preparation of ALCHNPs and encapsulation efficacy measurement

Chitosan (80%–85% deacetylation degree, Sigma Aldrich, USA) was dissolved in 0.3 % acetic acid (Glacial, Sigma Aldrich, USA) at 0.6 wt. % concentration at room temperature for 18 h. Then, allopurinol powder (≥ 99% HPLC, Selleckchem, USA) was added to the chitosan’s solution at 10 % w/w and further stirred for 6 h. Tripolyphosphate (Sigma Aldrich, USA) was dissolved in distilled water at 0.3 wt. % for 4 h. Then, tripolyphosphate’s solution was added to chitosan’s solution at 1:3 volume ratios under a strong agitation. The resulting solution was then centrifuged at 15,000 rpm for 45 min. The pellet was harvested and the allopurinol concentration in the supernatant was measured using a spectrophotometer at λ max 338 nm. Encapsulation efficacy was measured using the following equation.

Encapsulation efficacy (%) ═ (1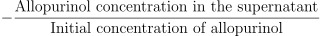) × 100

The pellet was harvested and lyophilized for 48 h. The produced powder was kept at 4 ^∘^C until use.

### Preparation of chitosan/alginate hydrogel, chitosan/alginate/ALCHNPs, and chitosan/alginate/CHNPs

Chitosan was dissolved in a 1% v/v acetic acid solution in distilled water containing 40 w/w % glycerol (Sigma Aldrich, USA) in a final concentration of 2 wt. % for 24 h. Then, the pH of the solution was adjusted to 7.0 by the drop-wise addition of 1 wt.% NaOH solution (Sigma Aldrich, USA). Sodium alginate (medium viscosity, Sigma Aldrich, USA) was dissolved in distilled water containing 40 w/w % glycerol at 1.5 wt. % for 12 h. Then, chitosan and sodium alginate solutions were mixed at 1:1 volume ratio and mixed for 6 h at room temperature. Finally, CHNPs or ALCHNPs were dispersed in the hydrogels at 15% w/w. The resulting composite hydrogel was lyophilized for 48 h for in vitro characterization.

### Scanning electron microscopy imaging

The microstructure of CHNPs, ALCHNPs, chitosan/alginate hydrogel, chitosan**/**alginate/ALCHNPs, and chitosan/alginate/CHNPs was evaluated after coating them with gold for 250 s. Scanning electron microscopy (SEM) imaging was performed under accelerating voltage of 26 kV.

### Release test

Release of allopurinol from chitosan**/**alginate/ALCHNPs was measured. Briefly, 100 mg of lyophilized chitosan**/**alginate/ALCHNPs hydrogels was immersed in 15 mL phosphate buffer saline (PBS) and kept at 37 ^∘^C for 144 h. At predetermined time points, release medium (0.5 mL) was taken, and its allopurinol content was measured by fitting the optical density values of the taken sample at λ max 338 nm into the standard curve of allopurinol in PBS. The same volume of fresh PBS was replenished after taking the test medium.

### Swelling assay

The swelling behavior of chitosan/alginate hydrogel, chitosan**/**alginate/ALCHNPs, and chitosan/alginate/CHNPs was studied as described before [18]. Briefly, 100 mg (M0) of each lyophilized hydrogel was immersed in 15 mL of normal saline solution. At different time points, the hydrogels were removed and immediately weighted (M1). The swelling behavior of scaffolds was calculated using the following equation:

Swelling (%) ═ (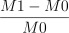) × 100

### Cell viability assay

Human dermal fibroblast cells were seeded onto the lyophilized chitosan/alginate, chitosan**/**alginate/ALCHNPs, and chitosan/alginate/CHNPs hydrogels at the density of 7000 cells per scaffold in a 96 well-plate and cultured with high glucose Dulbecco's Modified Eagle Medium (DMEM; Invitrogen, USA), 10% v/v fetal bovine serum (FBS; Invitrogen, USA), and 1% v/v antibiotics (Sigma Aldrich, USA) for 5 days. Cell viability assay was performed on days 1, 3, and 5 using an MTT assay kit (Abcam, USA). Cells in the empty plates served as the control group.

### Cell viability assay under oxidative stress

Human dermal fibroblast cells were seeded onto the lyophilized chitosan/alginate, chitosan**/**alginate/ALCHNPs, and chitosan/alginate/CHNPs hydrogels at the density of 7000 cells per scaffold in a 96 well-plate and cultured for 48 h. Then, 1% v/v H_2_O_2_ was added onto each well and incubated for 1 h. Finally, the cell viability assay was performed using an MTT assay kit (Abcam, USA). Cells without H_2_O_2_ and hydrogels served as the negative and positive control groups, respectively.

**Table 1:** Primer sets for the real time PCR assay

**Gene**	**Forward sequence (5′-3′)**	**Reverse sequence (5′-3′)**
Type I collagen	CCTCAGGGTATTGCTGGACAAC	CAGAAGGACCTTGTTTGCCAGG
Type II collagen	TGGTCTGCAAGGAATGCCTGGA	TCTTTCCCTGGGACACCATCAG
*NF-κB*	TACCATGCTGTTTGGTTCA	TCAAGCTACCAATGACTTTC
*GPx*	AGTTCGGACATCAGGAGAATGGCA	TCACCATTCACCTCGCACTTCTCA
*GAPDH*	GGGAAACTGTGGCGTGAT	AAAGGTGGAGGAGTGGGT

NF-κB: Nuclear factor κB; GPx: Glutathione peroxidase.

### Cell migration assay

Lyophilized chitosan/alginate, chitosan/alginate/ALCHNPs, and chitosan/alginate/CHNPs hydrogels were immersed in a high glucose DMEM for 5 days at 37 ^∘^C under gentle shaking. Then, the culture media was filtered. Human dermal fibroblast cells were seeded in a 24-well plate and cultured to reach 80% confluence. Then, a linear wound was made at the middle of the cellular monolayer using a 200 µL pipette tip. The cellular debris was removed by washing them with PBS. Then, cells were cultured with the hydrogels’ extract for 48 h. The wound size area was investigated for reduction using a light microscope and image analysis software.

### In vitro anti-inflammatory assay

The anti-inflammatory activity of the developed hydrogels was evaluated by their effects on interleukin 6 (IL-6) release from macrophage cells. Briefly, J774A1 cells (murine macrophage cell line) were seeded onto the lyophilized chitosan/alginate, chitosan/alginate/ALCHNPs, and chitosan/alginate/CHNPs hydrogels and cultured for 24 h. Then, each well was supplemented with 1 µg/mL lipopolysaccharide (Sigma Aldrich, USA) and incubated for another 12 h. Finally, IL-6, IL-1β, and tumor necrosis factor alpha (TNF-α) concentrations in each well were measured using an ELISA KIT (Sigma Aldrich, USA).

### Animal studies

Male Wistar rats (200–220 g, 10–12 weeks old) were used to induce intervertebral disk injury model. Animals were anesthetized by the intraperitoneal injection of ketamine and xylazine. A linear incision was made at the ventral plane of the animals’ tail using a surgical blade and C5-C6 caudal disk was exposed. Then, the intervertebral disk at this level was punctured to a depth of 3–5 mm using a 27 G needle and a slight negative pressure was applied to damage nucleus pulposus. The skin incision was then closed and animals were left to recover for two weeks. Animals were then re-anesthetized and divided into four groups (three animals in each group): 1) negative control group in which animals were treated with an injection of 2 µL PBS; 2) chitosan/alginate hydrogel group in which animals were treated with injection of 2 µL chitosan/alginate hydrogel; 3) chitosan/alginate hydrogel/CHNPs in which animals were treated with injection of 2 µL chitosan/alginate/CHNPs hydrogel; 4) chitosan/alginate hydrogel/ALCHNPs in which animals were treated with injection of 2 µL chitosan/alginate/ALCHNPs hydrogel. Eight weeks after the hydrogels injection, animals were sacrificed and the target intervertebral disks were harvested for histopathological examinations.

### Real-time PCR assay

Eight weeks after surgery, the animals were sacrificed and their intervertebral disks were harvested for the analysis of their tissue expression levels of type I collagen, type II collagen, nuclear factor kappa-B (NF-κB), and glutathione peroxidase (GPx) genes. After harvesting the intervertebral disk tissues, the RNA of the tissue samples was isolated using an RNA isolation kit (Thermofisher, USA). Then, cDNA strands were produced by a reverse transcription kit (Thermofisher, USA). Gene amplification of target genes was done by loading 3 µL of the cDNA samples into 17 µL of reaction master mix containing power cyber green (Thermofisher, USA). Finally, relative gene expression was calculated using 2 ^— ΔΔct^ method. Table 1 shows the primer sets for this experiment.

### 2.12a Ethical statement

The animal studies were performed in accordance with the U.K. Animals (Scientific Procedures) Act, 1986 and associated guidelines, EU Directive 2010/63/EU for animal experiments.

### Statistical analysis

Data were analyzed by GraphPad prism statistical analysis software using Student’s *t*-test and one-way analysis of variance (ANOVA).

## Results

### Microstructure studies

**Figure 1.**
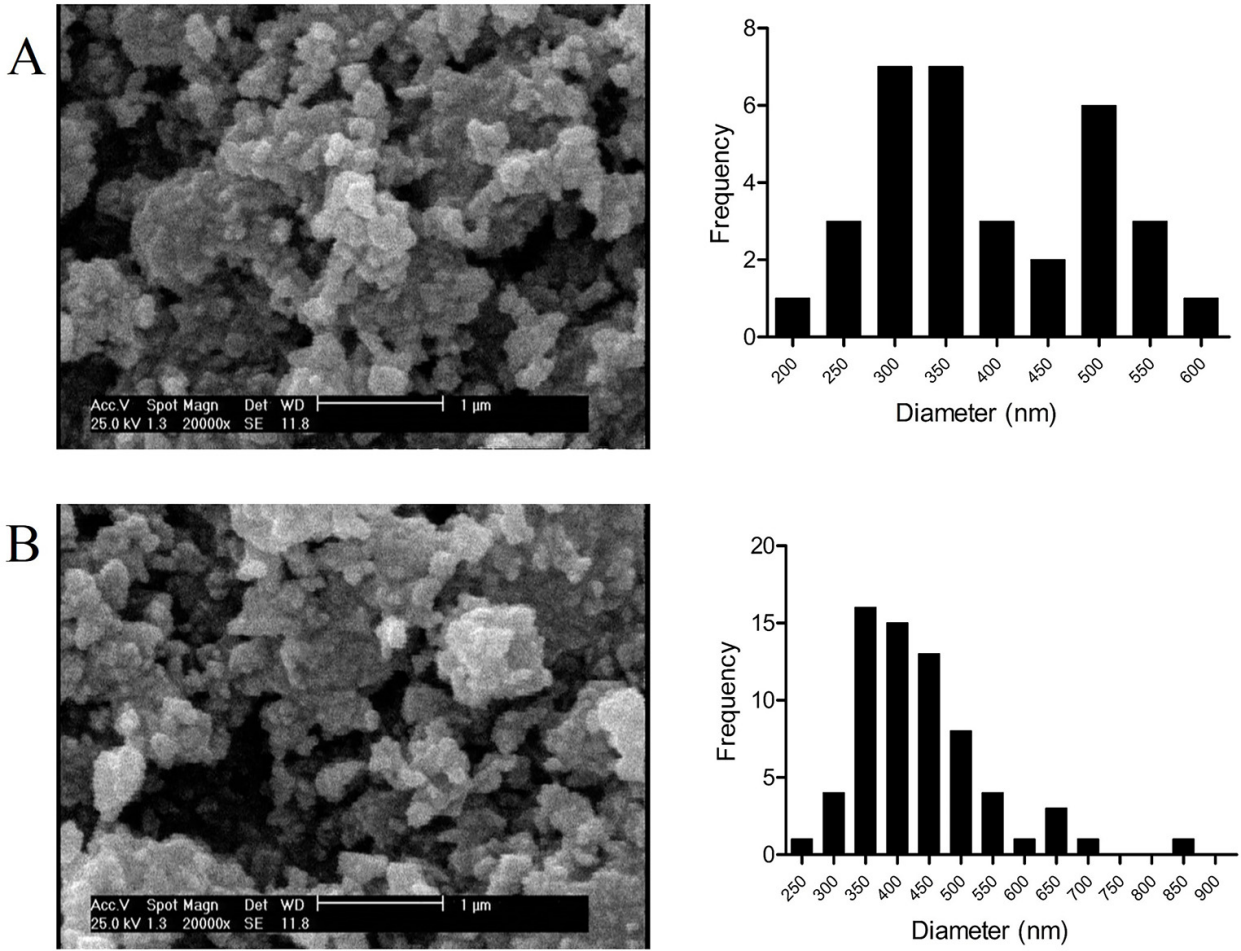
**Representative SEM images and particle size distribution of (A) CHNPs and (B) ALCHNPS.** SEM: Scanning electron microscopy; CHNPs: Chitosan nanoparticles; ALCHNPS: Allopurinol-incorporated CHNPs.

**Figure 2.**
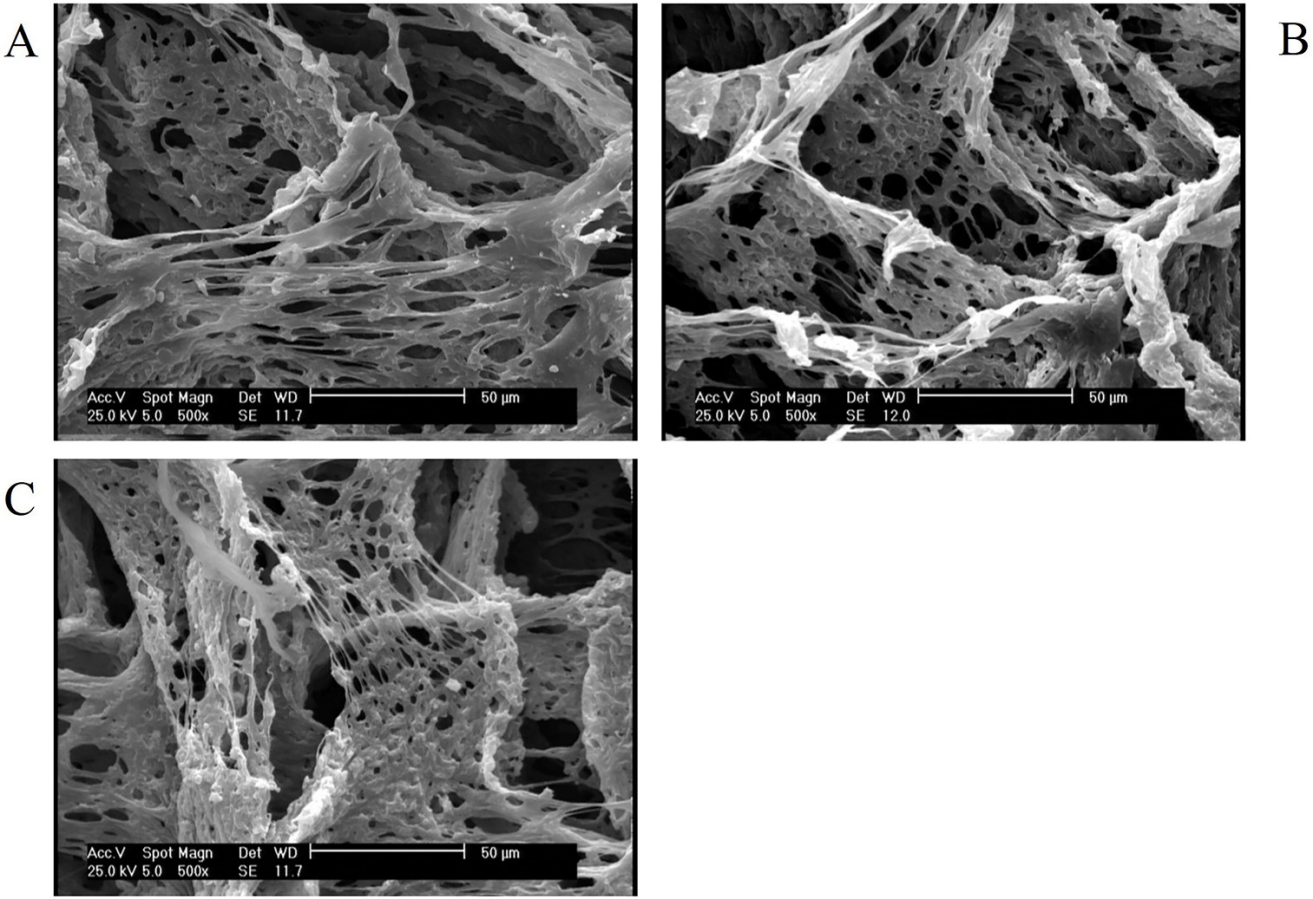
**Representative SEM images of lyophilized (A) chitosan/alginate, (B) chitosan/alginate/ALCHNPs, and (C) chitosan/alginate/CHNPs hydrogels.** SEM: Scanning electron microscopy; CHNPs: Chitosan nanoparticles; ALCHNPS: Allopurinol-incorporated CHNPs.

The SEM images (Figure 1) showed that ALCHNPs and CHNPs were round with a wide size distribution. Some of the particles were round and some of them were agglomerated. Particle size measurement using the ImageJ software showed that CHNPs and ALCHNPs had mean particle size of round 391.78 ± 104.59 nm and 437.10 ± 104.69 nm, respectively. Lyophilized chitosan/alginate, chitosan/alginate/ALCHNPs, and chitosan/alginate/CHNPs hydrogels (Figure 2) had a porous structure with the irregular shape of the pores. Furthermore, the size of the pores was not consistent among groups. The pore size measured in this research may not allow for cellular ingrowth. However, the hydrogels’ pore size and morphology are different from their lyophilized form.

### Release profile and encapsulation efficacy

**Figure 3.**
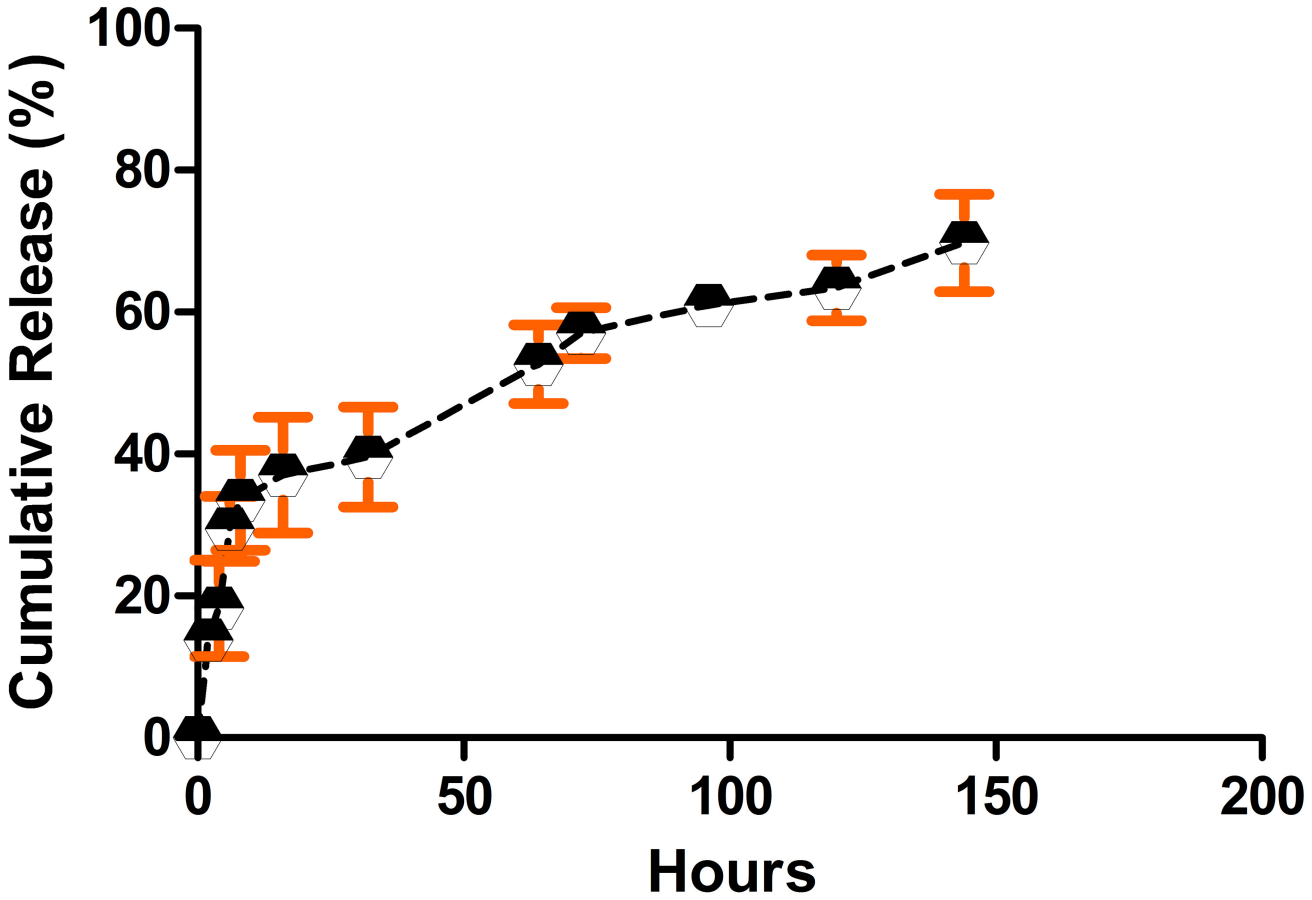
**The release profile of allopurinol from chitosan/alginate/ALCHNPs hydrogels.** The release test was conducted during a time course of 144 h. Data are presented as mean ± standard deviation. ALCHNPS: Allopurinol-incorporated chitosan nanoparticles.

Encapsulation efficacy measurement showed that encapsulation efficacy of allopurinol into ALCHNPs was about 46.99 ± 6.66%. Release test (Figure 3) showed that allopurinol was released from chitosan/alginate/ALCHNPs hydrogels in a sustained manner. At the initial hours, there was a fast release phase which was followed by a gradual increase in the rate of drug release.

### Swelling assay

**Figure 4.**
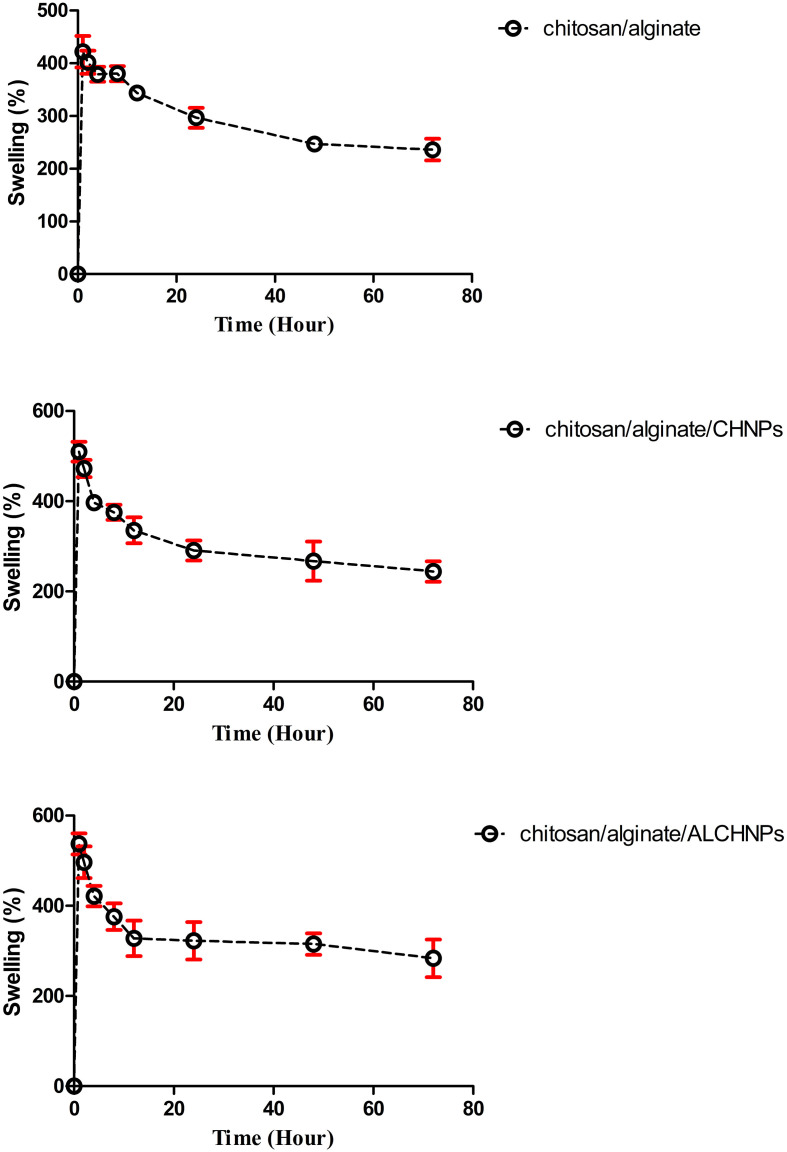
**The swelling behaviors of chitosan/alginate, chitosan/alginate/CHNPs, and chitosan/alginate/ALCHNPs hydrogels in PBS during the time course of 72 h.** Data are presented as mean ± standard deviation. CHNPs: Chitosan nanoparticles; ALCHNPS: Allopurinol-incorporated CHNPs; PBS: Phosphate buffer saline.

Swelling assay (Figure 4) showed that all hydrogels had the highest rate of swelling in the first hour of immersion in PBS. After this time point, the percentage of swelling had a decreasing trend. In the first hour, the swelling percentage for chitosan/alginate, chitosan/alginate/CHNPs, and chitosan/alginate/ALCHNPs hydrogels was 421.94 ± 29.75%, 509.78 ± 21.80%, and 537.06 ± 23.35%, respectively.

### Cell viability assay

**Figure 5.**
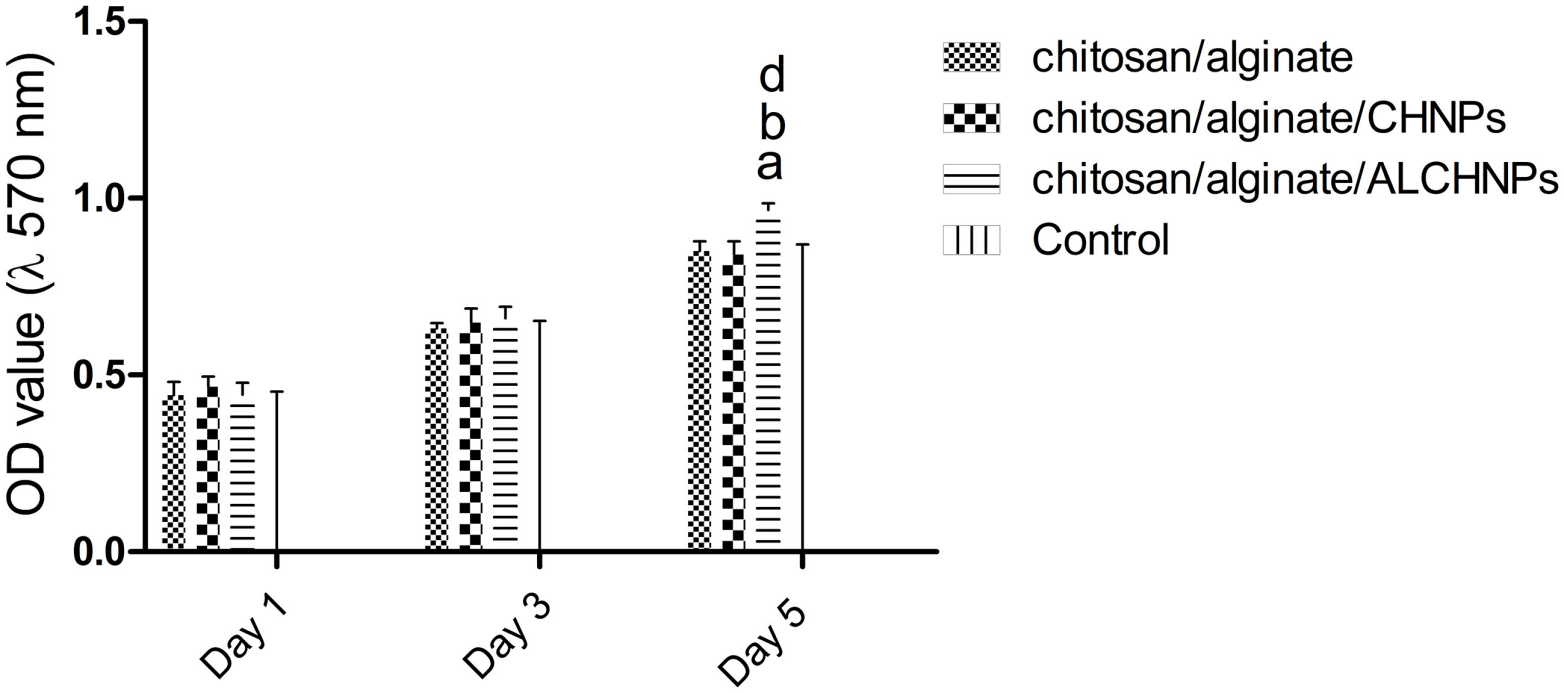
**Histograms comparing the viability of human dermal fibroblast cells cultured on chitosan/alginate, chitosan/alginate/CHNPs, and chitosan/alginate/ALCHNPs hydrogels.** Control groups are the cells cultured on tissue culture plate. a, b, and d showing the *P* value <0.05 relative to chitosan/alginate, chitosan/alginate/CHNPs, and control groups, respectively. Data are presented as mean ± standard deviation. CHNPs: Chitosan nanoparticles; ALCHNPS: Allopurinol-incorporated CHNPs.

The MTT assay (Figure 5) showed that on days 1 and 3, the difference between chitosan/alginate, chitosan/alginate/CHNPs, and chitosan/alginate/ALCHNPs and control groups was not statistically significant (*P* > 0.05). On day 5, chitosan/alginate/ALCHNPs group had significantly higher cell viability compared with other groups, indicating higher metabolic activity of the cells in this group.

### Cell viability assay under oxidative stress

**Figure 6.**
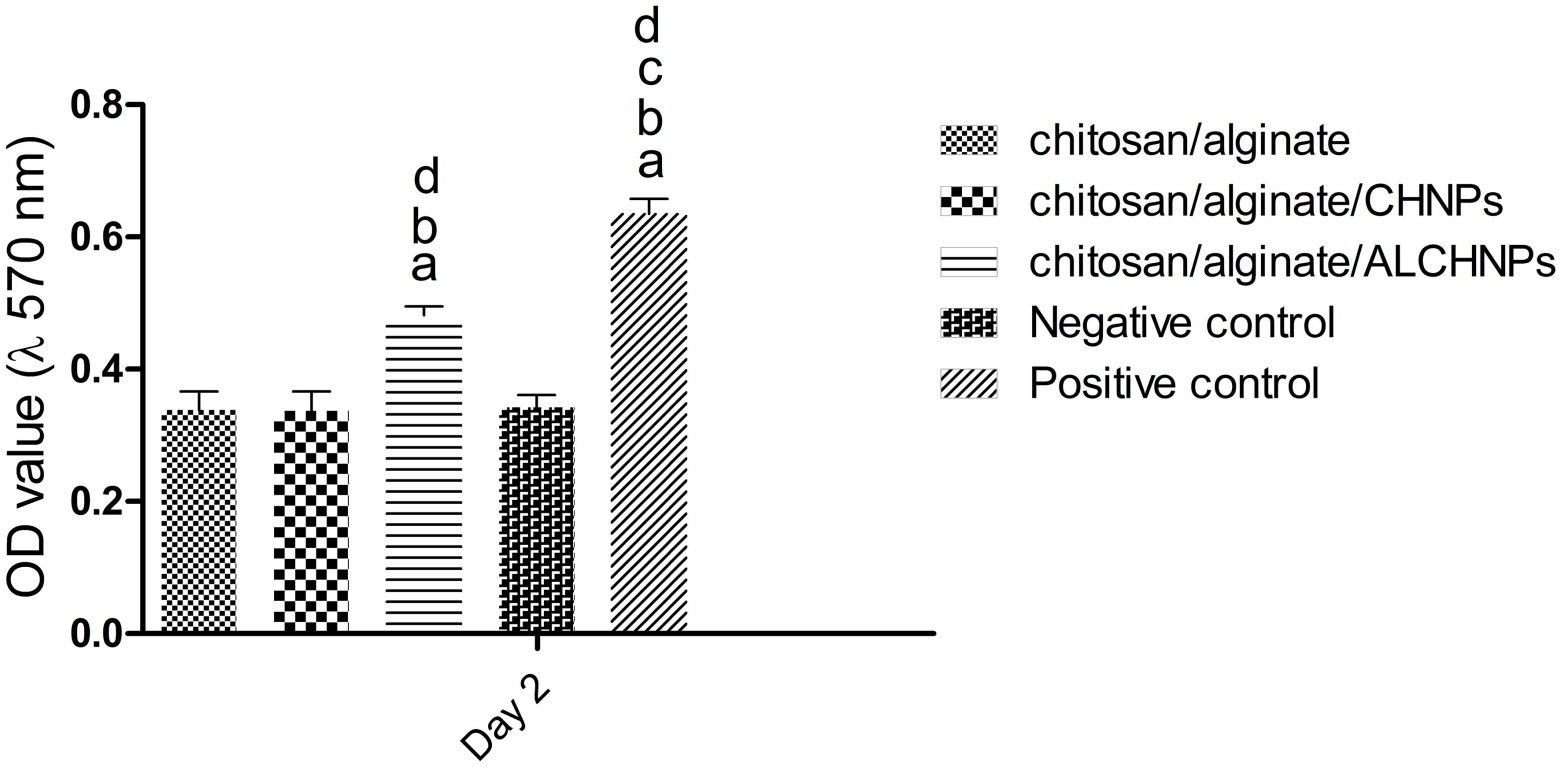
**Histograms comparing the viability of human dermal fibroblast cells cultured on the chitosan/alginate, chitosan/alginate/CHNPs, and chitosan/alginate/ALCHNPs hydrogels in the presence of 1% v/v H_2_O_2_.** Negative and positive control groups are the cells cultured on tissue culture plate with and without H_2_O_2_, respectively. a, b, c, and d show the *P* value <0.05 relative to chitosan/alginate, chitosan/alginate/CHNPs, chitosan/alginate/ALCHNPs, and negative control groups, respectively. Data are presented as mean ± standard deviation. CHNPs: Chitosan nanoparticles; ALCHNPS: Allopurinol-incorporated CHNPs.

The MTT assay under oxidative stress (Figure 6) showed that cells cultured on chitosan/alginate/ALCHNPs hydrogels had significantly higher cell viability than the cells cultured on chitosan/alginate/CHNPs and chitosan/alginate hydrogels (*P* < 0.05). Therefore, cells have been more resistant against oxidative stress in the allopurinol-loaded hydrogel group.

### Cell migration assay

**Figure 7.**
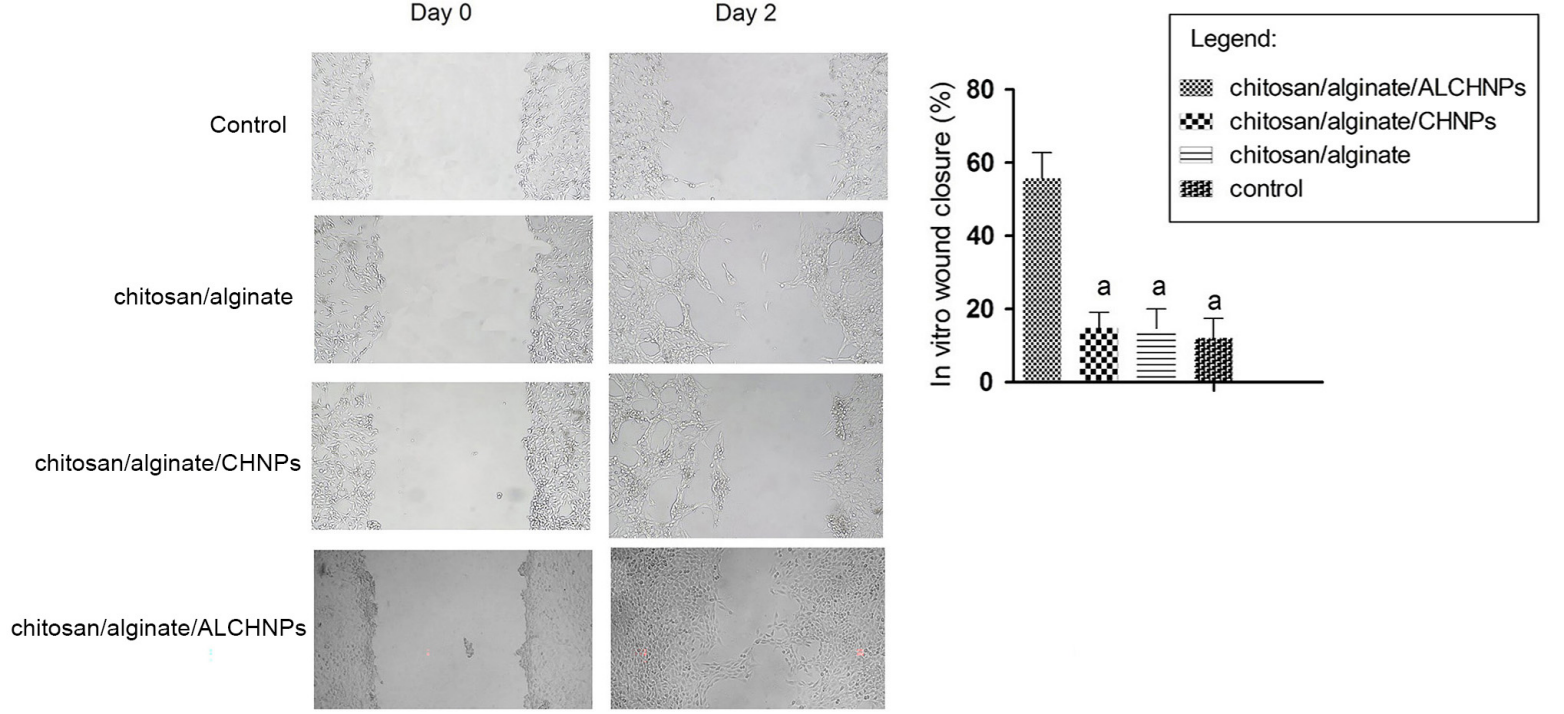
**The in vitro wound closure assay with human dermal fibroblast cells cultured with the extract of different hydrogels.** The histogram demonstrates the percentage of wound closure on day 2 after the scratch test. a shows the *P* value <0.05 relative to chitosan/alginate/ALCHNPs group. Data are presented as mean ± standard deviation. CHNPs: Chitosan nanoparticles; ALCHNPS: Allopurinol-incorporated CHNPs.

In vitro wound closure assay (Figure 7) showed that cells cultured with the extract of chitosan/alginate/ALCHNPs hydrogels had significantly higher percentage of in vitro wound closure compared with other groups (*P* < 0.05). As shown in the light microscopy images, the number of fibroblast cells in the scratch area was obviously higher in the cells cultured with the extract of chitosan/alginate/ALCHNPs hydrogels.

### In vitro anti-inflammatory assay

**Figure 8.**
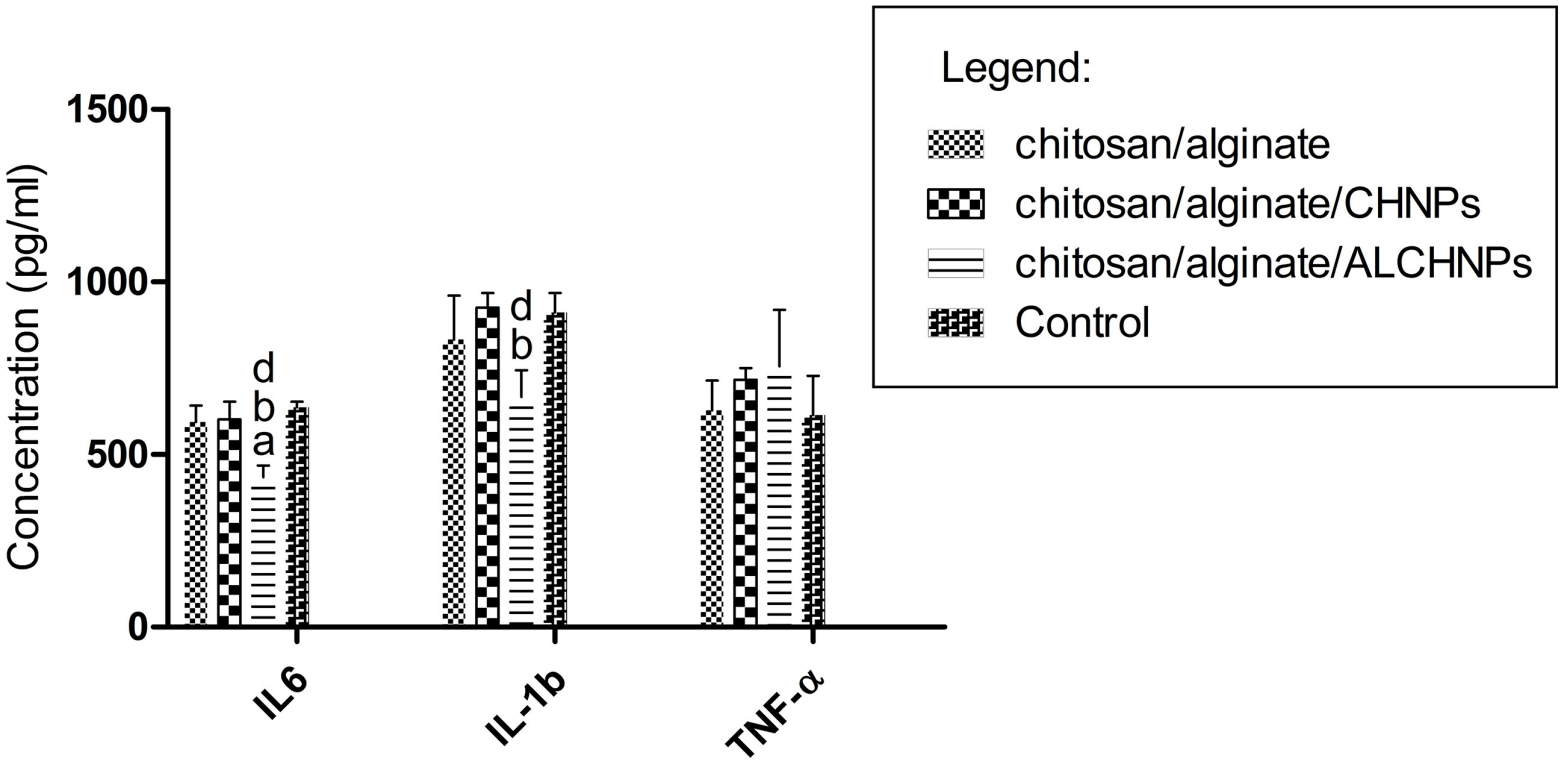
**Concentration of IL-6, IL-1β, and TNF-α in supernatant of macrophage cells cultured on different lyophilized hydrogels.** a, b, and d show the *P* value <0.05 relative to chitosan/alginate, chitosan/alginate/CHNPs, and control groups, respectively. Data are presented as mean ± standard deviation. CHNPs: Chitosan nanoparticles; ALCHNPS: Allopurinol-incorporated CHNPs; IL: Interleukin; TNF-α: Tumor necrosis factor alpha.

The anti-inflammatory assay (Figure 8) showed that the macrophage cells cultured on chitosan/alginate/ALCHNPs hydrogels released significantly lower amount of IL-6 compared with the cells cultured on other hydrogels and the cells cultured on tissue culture plate (*P* < 0.05). The concentration of IL-1β in the supernatant of macrophage cells in the chitosan/alginate/ALCHNPs group was significantly lower than in the chitosan/alginate/CHNPs and control groups (*P* < 0.05). However, no statistically significant difference was found in TNF-α concentration between the groups (*P* > 0.05).

### Animal studies

**Figure 9.**
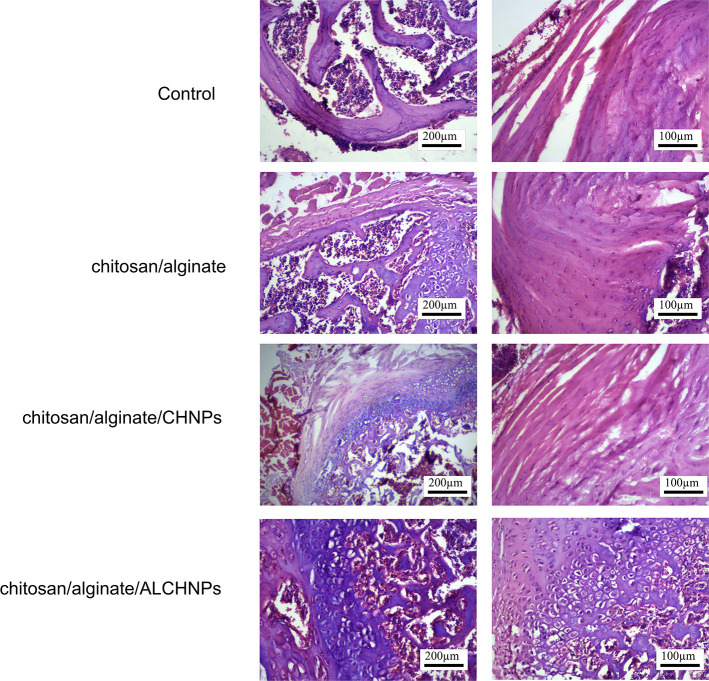
**The representative hematoxylin eosin staining images of nucleus pulposus in rats treated with different hydrogels; control groups are the animals treated with PBS injection.** CHNPs: Chitosan nanoparticles; ALCHNPS: Allopurinol-incorporated CHNPs; PBS: Phosphate buffer saline.

In the control group, histopathological examinations (Figure 9) showed that the nucleus pulposus was replaced by fibrous tissue, and disk degeneration was obvious. In chitosan/alginate and chitosan/alginate/CHNPs groups, the fibrous tissue was decreased and the nucleus pulposus could slightly restore its structure. In the chitosan/alginate/ALCHNPs group, the defect site had been filled with ECM components and fibrous tissue was minimal.

**Figure 10.**
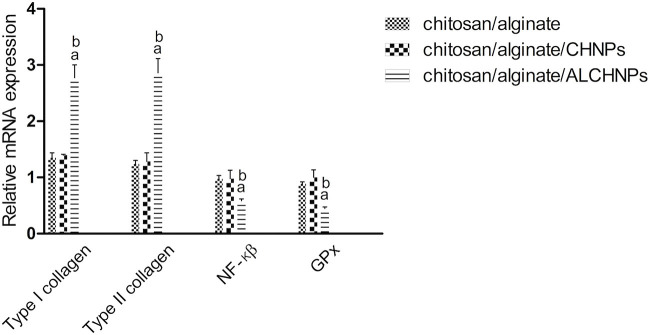
**The gene expression profile of different genes in intervertebral disk of rats treated with hydrogel systems.** a and b show the *P* value <0.05 relative to chitosan/alginate and chitosan/alginate/CHNPs groups, respectively. Gene expression levels were quantified relative to control group. Data are presented as mean ± standard deviation. CHNPs: Chitosan nanoparticles; ALCHNPS: Allopurinol-incorporated CHNPs; GPx: Glutathione peroxidase; NF-κB: Nuclear factor kappa-B.

The relative expression levels of type I collagen, type II collagen, NF-κB, and GPx genes in the allopurinol-loaded hydrogel group were 2.77 ± 0.2%, 2.86 ± 0.25%, 0.58 ± 0.03%, and 0.45 ± 0.02%, respectively. Gene expression studies (Figure 10) showed that chitosan/alginate/ALCHNPs hydrogels significantly promoted the tissue expression levels of type I collagen and type II collagen genes compared with other groups (*P* < 0.05). Furthermore, the expression levels of NF-κB and GPx genes were significantly lower in the chitosan/alginate/ALCHNPs group than in the other groups (*P* < 0.05).

## Discussion

Intervertebral disk degeneration imposes a pressure on nerve roots and may lead to the chronic pain in the extremities and the spinal column. Although significant progress has been made, the current treatments have palliative effects only [19]. Therefore, emerging technologies, such as tissue engineering, have been developed to address the current challenges. In this framework, hydrogel-based drug and cell delivery systems were widely explored for intervertebral disk regeneration [7].

In the current research, a composite hydrogel system was developed for sustained delivery of allopurinol into the nucleus pulposus. As it is shown in the microstructure studies, the nanoparticles have a small size that may facilitate drug internalization into the nucleus pulposus cells. Furthermore, as these particles have a net positive charge, they can attach to the negatively charged plasma membrane of nucleus pulposus cells and so increase the drug’s bioavailability [20]. The pores observed in the SEM images of lyophilized hydrogels could be due to the evaporation of the solvent in the freeze drying process [21]. The high encapsulation efficacy is one of the main advantages of CHNPs in drug delivery applications. Encapsulation of allopurinol in ALCHNPs may be due to the hydrophobic interactions, electrostatic forces, or simply due to physical entrapment of allopurinol within the polymeric chains of chitosan [22]. Drug release assay confirmed a sustained release profile of allopurinol from chitosan/alginate/ALCHNPs hydrogel. The release of allopurinol from these composite systems may be due to the swelling of chitosan’s matrix in the ALCHNPs or due to the diffusion mechanism. In addition, biodegradation may have also released allopurinol from the matrix of the composite hydrogel [23].

The swelling capacity of the hydrogel systems is crucial for water retention after injection into the nucleus pulposus. This study showed that the swelling percentage of the hydrogel scaffolds started to level off following the first hour of immersion in an aqueous solution that could be due to the biodegradation of the hydrogels. Due to weak inter-chain bonding of chitosan and alginate, the hydrogel produced from the combination of chitosan and alginate is highly prone to biodegradation [15, 24]. Despite being a safe cross-linking method, polyelectrolyte complexation does not provide durable scaffolding systems. This drawback can be addressed by using chemical cross-linking reagents. However, their potential cytotoxic effects need to be investigated.

The biocompatibility of tissue-engineered constricts is essential for their successful clinical translation [25]. As it is shown in the MTT assay, our developed hydrogels were compatible with fibroblast cells and promoted their metabolic activity. Further, chitosan/alginate/ALCHNPs successfully protected fibroblast cells against oxidative stress. It could be that allopurinol may have quenched free radicals produced by H_2_O_2_ and protected cells against peroxidation. In accordance with this research, Luo et al. [26] showed that allopurinol protected rat cardiomyocytes against oxidative stress by regulating Nrf2/p62. Yang et al. [27] showed that allopurinol protected endothelial cells against oxidative stress in a rabbit model of alloxan-induced diabetes mellitus.

The chemotic effect of hydrogel systems is beneficial for the repair of nucleus pulposus injury. Cells around the bioactive hydrogel may migrate toward the hydrogel and grow into its matrix. This study showed that fibroblast cells cultured with the extract of chitosan/alginate/ALCHNPs had significantly higher migratory activity than the cells cultured with the extract of other hydrogels. It could be that allopurinol may have increased the migration of fibroblast cells. However, little data is available in the literature to explain this result.

Chronic inflammation exacerbates the nucleus pulposus injury and damages its ECM. Therefore, the immunomodulatory activity of hydrogels may be of therapeutic value in treating nucleus pulposus injury. Our results revealed that macrophage cells cultured on chitosan/alginate/ALCHNPs hydrogels released significantly lower amount of IL-6 and IL-1β than other hydrogels, indicating that incorporation of allopurinol has imparted anti-inflammatory activity to the hydrogel system. In this regard, Wang et al. [28] showed that allopurinol inhibited the activation of NLRP3 inflammasome and alleviated inflammatory responses in the liver of diabetic rats. Furthermore, Wang et al. [29] showed that allopurinol reduced oxidative stress and inflammation in the renal cortex of fructose-fed rats by microRNA-377.

Animal studies showed that the healing function of chitosan/alginate/ALCHNPs hydrogels was highest among other groups. This higher healing function may be due to the following reasons. Collagen type II and I are the major constituents of nucleus pulposus and their upregulation via chitosan/alginate/ALCHNPs hydrogels implies that this delivery system may have increased ECM synthesis in the injured nucleus pulposus. In addition, the downregulation of NF-κB via chitosan/alginate/ALCHNPs hydrogel may also imply that this hydrogel has modulated inflammatory responses in the nucleus pulposus via PI3K/AKT/NF-κB signaling pathway [30, 31]. However, the expression profile of other inflammation-associated genes should be investigated to substantiate this theory. *GPx* gene is activated in oxidative stress conditions and results in alleviation of oxidative stress [32]. As it is shown in the gene expression studies, tissue expression levels of this gene were lowest in the chitosan/alginate/ALCHNPs hydrogel group among other groups, implying that this hydrogel has modulated oxidative stress in the injured nucleus pulposus.

These results suggest the potential use of chitosan/alginate/ALCHNPs hydrogels to treat the intervertebral disk injuries in the clinic. We showed for the first time that allopurinol-loaded hydrogel promoted intervertebral disk repair that could be due to its potential to modulate oxidative stress, reduce inflammation, and improve matrix synthesis.

## Conclusion

In this research, allopurinol was loaded into the CHNPs and then dispersed into the chitosan/alginate hydrogel system to treat intervertebral disk injury in a rat model. In vitro studies showed that the composite hydrogel had a porous structure and promoted the viability and migration of human dermal fibroblast cells. Animal studies showed that allopurinol-delivering hydrogels had a significantly higher healing effect on injured nucleus pulposus than other experimental groups. Gene expression studies implied that the hydrogel system may have increased the ECM synthesis, alleviated inflammatory responses, and modulated the inflammation.
